# Association of peripheral CD8+ T cell activation with disease activity and treatment resistance in systemic lupus erythematosus

**DOI:** 10.1136/rmdopen-2024-005122

**Published:** 2025-02-26

**Authors:** Yuya Fujita, Shingo Nakayamada, Satoshi Kubo, Yusuke Miyazaki, Koshiro Sonomoto, Hiroaki Tanaka, Yoshiya Tanaka

**Affiliations:** 1 First Department of Internal Medicine, University of Occupational and Environmental Health, Kitakyushu, Japan; 2 Department of Molecular Targeted Therapies (DMTT), University of Occupational and Environmental Health, Kitakyushu, Japan; 3 Department of Clinical Nursing, School of Health Sciences, University of Occupational and Environmental Health, Kitakyushu, Japan; 4 The First Department of Internal Medicine, School of Medicine, University of Occupational and Environmental Health, Kitakyushu, Japan

**Keywords:** Lupus Erythematosus, Systemic, Autoimmune Diseases, T-Lymphocytes

## Abstract

**Objective:**

Various immune-cell subsets intricately mediate the pathogenesis of systemic lupus erythematosus (SLE). However, the role of CD8^+^ T cells in SLE remains unclear. We investigated the proportions and characteristics of peripheral CD8^+^ T cells and their association with clinical manifestations of SLE.

**Methods:**

We retrospectively enrolled 211 patients with SLE and 48 age- and sex-matched healthy controls (HCs). Peripheral CD8^+^ T cells were analysed using flow cytometry. The primary endpoint was the comparison of peripheral CD8^+^ T cell subset characteristics between patients and HCs.

**Results:**

Patients with SLE (mean age, 42.3 years; women, 89% and mean disease duration, 112.8 months) had significantly higher proportions of naïve CD8^+^ T cells (CCR7^+^CD45RA^+^), CD8^+^ terminally differentiated effector memory cells (CCR7^−^CD45RA^+^) and activated CD8^+^ T cells (CD38^+^HLA-DR^+^) in peripheral blood mononuclear cells than HCs (p<0.001). Activated CD8**^+^** T cells produced granzyme B and interferon-γ, which correlated with serum double-stranded (ds) DNA antibodies (rs=0.3146, p<0.0001) and 50% haemolytic unit of complement (rs=−0.3215, p=0.0003), and were significantly increased in patients with active systemic, renal or haematological involvement (p<0.05). Cluster analysis-based subgroup classification based on CD8 cell differentiation and activation revealed a group with high numbers of activated CD8^+^ T cells, highly active SLE and organ damage, including active nephritis and persistently high cell counts after a 24-week treatment, indicating treatment resistance (high anti-dsDNA antibody titres and high glucocorticoid doses).

**Conclusion:**

In SLE, greater proportions of highly cytotoxic and proinflammatory activated CD8^+^ T cells in peripheral blood-modulated disease activity, organ damage and residual treatment resistance, presenting a potential treatment target.

**WHAT IS ALREADY KNOWN ON THIS TOPIC**

- A high proportion of CD8^+^ T cells are present in the organ tissues of patients with systemic lupus erythematosus (SLE). The function of CD8^+^ T cells and their association with clinical symptoms have not yet been reported.

**WHAT THIS STUDY ADDS**

- The study performed peripheral blood immunophenotyping and analysed clinical signs in 211 patients with SLE and found that the percentage of activated CD8^+^ T cells in their peripheral blood was associated with disease activity, organ damage and resistance to treatment.

**HOW THIS STUDY MIGHT AFFECT RESEARCH, PRACTICE OR POLICY**

- This study may provide a basis for research into activated CD8^+^ T cells as a new surrogate marker and therapeutic target.

## Introduction

 Systemic lupus erythematosus (SLE) is associated with multiorgan inflammation and the production of characteristic autoantibodies, such as anti-double-stranded (ds) DNA and anti-Sm antibodies.^1^ Various immunosuppressants are used in SLE treatment.^2^ Recently, belimumab (an antibody of the anti-B cell activating factor from the tumour necrosis factor family^3^) and anifrolumab (an anti-interferon (IFN) α-antibody^4^) were used as novel treatment modalities that target immune abnormalities in SLE. However, SLE is often difficult to treat in clinical practice, owing to treatment resistance, adverse events or recurrent relapse.

The complex involvement of abnormal diverse immune-cell subsets in SLE pathogenesis is a major contributor to the increased difficulty in treating SLE.^5 6^ We previously reported that the CXC chemokine receptor (CXCR) 5^+^CXCR3^+^ B cell lymphoma (Bcl) 6^+^T-bet^+^ interleukin (IL)−21^+^ IFN-γ^+^ T (T follicular helper (Tfh)/T helper (Th) 1) cells modulate the differentiation of autoantibody-producing B cells via IL-21/IFN-γ production, and their expression is increased in the peripheral blood of patients with SLE.^7^ This increase disrupts the balance between Tfh cells (which induce inflammation) and follicular regulatory T cells (which suppress differentiation into Tfh cells),^8^ and the increased proportion of Tfh cells in the peripheral blood is responsible for the resistance to SLE treatment.^9^

In contrast to the multitude of reports on CD4^+^ T cells, there are few reports on CD8^+^ T cells. Recently, single-cell Ribonucleic Acid (RNA) sequencing from renal tissue samples obtained from patients with lupus nephritis revealed abundant CD8^+^ T cells in renal tissues, and this cell type is a major IFN-γ-producing cell subset that is important for inducing inflammation.^10^ Furthermore, cytotoxic CD8^+^ T cells in renal tissue can damage the glomeruli and interstitium and are associated with treatment resistance and progression to end-stage renal failure.^11 12^ However, these previous reports on the involvement of CD8^+^ T cells in the inflammatory pathology of SLE are exclusively based on the examination of renal tissue samples and had limited sample sizes. Several studies have suggested that peripheral immunophenotypes reflect the tissue’s pathological conditions,^12^,^14^ and peripheral blood samples can be collected in an easy and minimally invasive manner. Thus, peripheral blood samples can be investigated in large-scale studies, and their application in real-world clinical practice is anticipated. Herein, we investigated the association of the characteristics of peripheral CD8^+^ T cells in patients with SLE who showed clinical manifestations of SLE. Thereby, the aim of this study was to identify subsets of CD8^+^ T cells and their function in relation to the pathogenesis of SLE and provide new approaches to novel therapies for SLE.

## Patients and methods

### Participants

The Hospital of the University of Occupational and Environmental Health, Fukuoka, Japan and its affiliated institutions are involved in the medical care of all patients with SLE in the region. Among these patients, those who were hospitalised for treatment in the First Department of Internal Medicine and provided written informed consent were registered in the LOOPS registry.^15 16^ In this study, we extracted data from the LOOPS registry on 211 East Asian patients who were initially admitted to our hospital between November 2012 and December 2018 and diagnosed with SLE according to the classification criteria of the 2012 Systemic Lupus International Collaborating Clinics (SLICC)^17^ or the 2019 European Alliance of Associations for Rheumatology/American College of Rheumatology (ACR).^18^ We enrolled 48 age- and sex-matched healthy controls (HCs) as the control group. Before the initiation of a new treatment, peripheral blood samples (20 mL) were collected. Immunophenotyping was only performed in patients who had completed 24 weeks of treatment and had provided consent. Written informed consent was obtained from the HCs and patients with SLE in accordance with the Declaration of Helsinki. This study was approved by the University of Occupational and Environmental Health Ethics Committee, Japan (UOEHCRB21-069 and UOEHCRB21-103).

### Data of patients with SLE and evaluation methods

Clinical data at baseline and at 6 months were collected retrospectively from the medical records. Disease activity was evaluated using the Safety of Estrogens in Lupus Erythematosus National Assessment SLE Disease Activity Index (SELENA-SLEDAI),^19^ and organ involvement was evaluated using the British Isles Lupus Assessment Group (BILAG) index^20 21^ and the International Society of Nephrology/Renal Pathology Society classification for lupus nephritis.^22^ Laboratory tests included the white blood cell (WBC) count, lymphocyte count, C reactive protein (CRP) levels, erythrocyte sedimentation rate (ESR), serum immunoglobulin G (IgG), anti-dsDNA antibody, anti-Sm antibody and 50% haemolytic unit of complement (CH_50_).

### Study design

In this retrospective observational study, the primary endpoint was the comparison of characteristics of peripheral CD8^+^ T cell subsets among the participants with SLE and the HCs. The secondary endpoints included the association between clinical parameters of SLE and percentages of peripheral CD8^+^ T cell subsets, the stratification of patients with SLE according to peripheral CD8^+^ T cell subsets and the association among clinical parameters after 24 weeks and percentages of peripheral CD8^+^ T cell subsets. Immunophenotyping after 24 weeks of treatment was only performed in patients who completed the full 24-week treatment and gave consent.

### Sample size calculation

A sample size of 45 cases each was calculated to achieve 90% power with an alpha value of 0.05 based on the results of previous observational studies and assuming a 2% difference in peripheral blood immunophenotyping between patients with SLE and HCs.^9^

### Flow cytometric analysis

**Figure 1:**
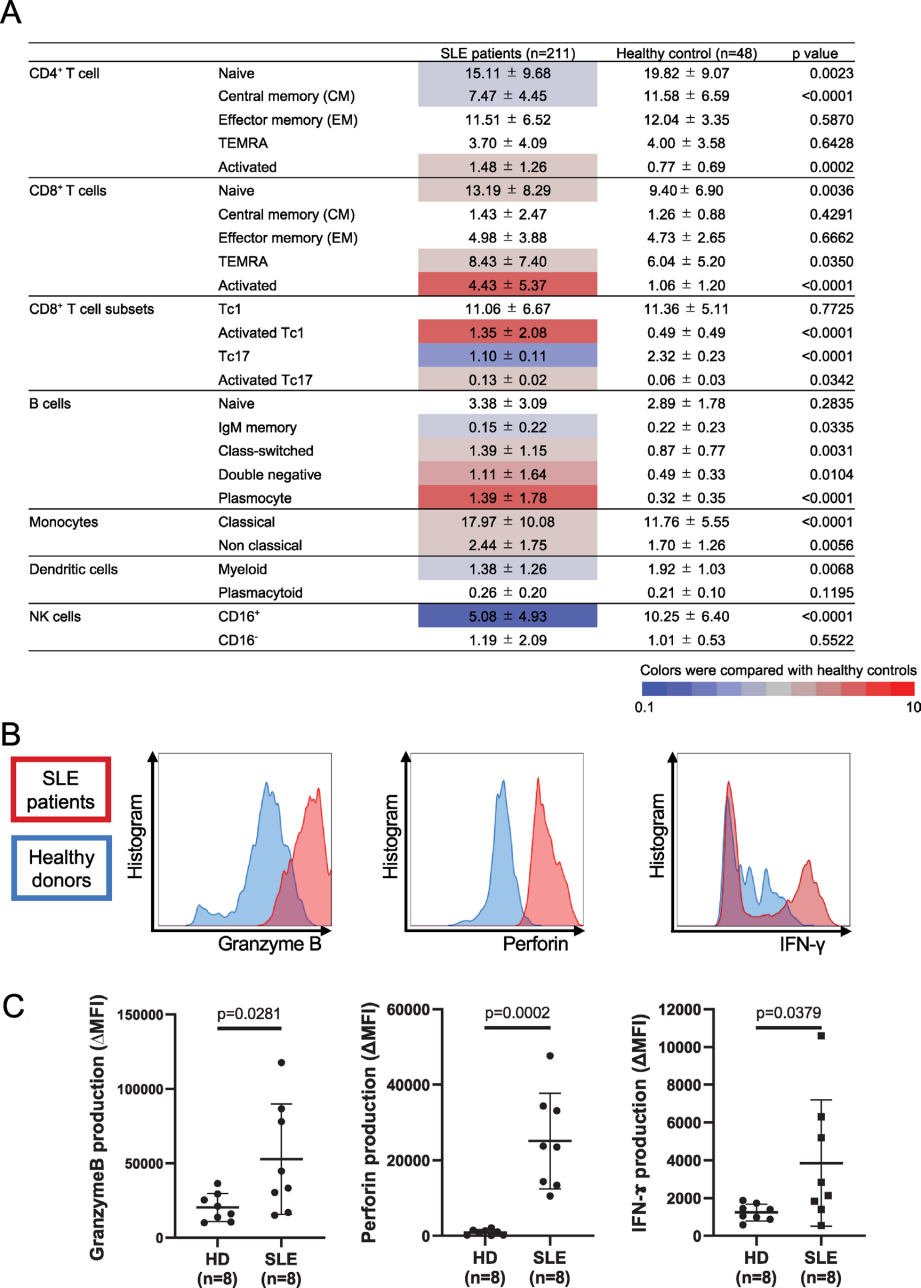
Differences in the percentage of peripheral blood lymphocyte subsets and function between patients with SLE and HCs. (**A**) Phenotypes of lymphocytes, monocytes, dendritic cells and NK cells in peripheral blood. Values represent mean±SD percentage, with levels that were significantly different in the patient group compared with those in the HC group, highlighted in colour (blue for decrease and red for increase). *P* values in the univariate analysis were determined using student’s t-test. TEMRA: terminally differentiated effector memory cells and Tfh: T follicular helper cells. (**B) and (C**) Granzyme B, perforin and IFN-γ expression in CD8^+^CD38^+^ HLA DR^+^ T cells from patients with SLE and HCs. (****Figure** B**) shows a histogram that is representative of a single sample of patients with SLE and HCs (SLE: red and HC: blue). (****Figure** C**) displays the MFI of each participant. The Mann–Whitney U test was used to determine statistical significance. HCs, healthy controls; HD, healthy donor ;HLA, human leucocyte antigen; IFN-γ, interferon-γ; IgM, immunoglobulin M; IVCY, intravenous cyclophosphamide; MFI, mean fluorescence intensity; NK, natural killer; RTX, rituximab; SLE, systemic lupus erythematosus; Tfh, T follicular helper.

All patients enrolled in this study were registered in the FLOW registry. Before initiation of new treatment, peripheral blood mononuclear cells (PBMCs) were collected from peripheral blood and immunophenotyped by multicolour flow cytometry (FACSVerse Cell Analyser, BD Biosciences, Franklin Lakes, NJ, USA). The results were recorded in the registry. PBMCs were isolated from peripheral blood samples using lymphocyte separation medium (ICN/Cappel, Costa Mesa, CA, USA), resuspended in phosphate-buffered saline (PBS) or 3% human IgG (Baxter International, Deerfield, IL, USA) to block Fc receptors and prevent non-specific antibody binding and incubated for 15 min at 4 °C in the dark. The cells were washed with PBS containing 1% bovine serum albumin. Background fluorescence was assessed using appropriate isotype- and fluorochrome-matched control monoclonal antibodies. After staining with antibodies (described inonline supplemental table 1), the cells were assessed by multicolour flow cytometry and the data were analysed with FlowJo (Tree Star, Ashland, OR, USA). The phenotype of immune-cell subsets was defined based on the Human Immunology Project protocol of comprehensive eight-colour flow cytometric analysis proposed by the National Institutes of Health/Federation of Clinical Immunology Societies,^23^ with some modifications for detecting Tfh cells.^24^ Details of the gating strategy are shown in onlinesupplemental figure 1  2.

For intracytoplasmic staining, cells were stimulated for 5 hours with phorbol 12-myristate 13-acetate (50 ng/mL), ionomycin (1 µg/mL) and brefeldin A (2.5 µg/mL). After staining with cell surface markers for 30 min at 4 °C, cells were washed with Fluorescence Activated Cell Sorter (FACS) buffer and fixed for 30 min at 4 °C with transcription factor buffer (BD Biosciences Pharmingen, San Diego, CA, USA) and then Perm/Wash solution (BD Biosciences).

### Statistical analysis

JMP Pro 15.2.0 (Cary, NC, USA) was used for statistical and cluster analyses and to generate heatmaps. Continuous data are expressed as the mean±SD and categorical data as the frequency with proportion (%). The significance of intergroup differences was evaluated using student’s t-test. Spearman’s rank correlation coefficient was used for correlation analysis. Cluster analysis was performed using Ward’s method^25^ to minimise the total within-cluster variance, with Euclidean distance as the dissimilarity measure. The analysis incorporated the immunophenotypes of CD8^+^ T cells of patients with SLE as clustering factors. The number of clusters was determined based on the evaluation of profile plots, where the cut-off point was identified as a sudden increase in distance, resulting in division into three distinct clusters. Statistical significance was defined as p value<0.05. Missing data were compensated using JMP’s Automated Data Imputation.

## Results

### Clinical characteristics of patients with SLE at baseline

The baseline characteristics of patients with SLE and HCs are shown in table 1. Among the patients with SLE, the mean age was 42 years, and 89.6% were women; the mean disease duration was 43 months. The median baseline glucocorticoid dose was 0 mg prednisolone (PSL) equivalent, and most patients with SLE were not administered PSL. Hydroxychloroquine has been available for prescription in Japan since July 2015, with a low concomitant rate of 10.0%. The rate of concomitant use of immunosuppressants was 33.2%; moreover, 42.7% of patients with SLE were untreated. The serological tests revealed that antibody titres of anti-dsDNA and anti-Sm were 89.9 and 46.4 U/mL, respectively, whereas the CH50 level was 37.3 U/mL. Assessment of disease activity revealed that the SELENA-SLEDAI score was 17.2, and patients with at least one BILAG index A organ-domain involvement or at least two BILAG index B organ-domain involvements accounted for 66.4% of the SLE subcohort. Many cases of newly diagnosed and untreated, highly disease-active SLE were included in this study.

**Table 1:** Baseline clinical characteristics of the patients with SLE and HCs

Background factors	Patients with SLE (n=211)	HCs (n=48)	P value
Average age	42.3±16.3	46.4±15.5	0.1061
Female, *n* (%)	189 (89.6)	42 (87.5)	0.676
Disease duration (month)	43 (3; 163)		
Treatment naive (%)	90 (42.7)		
Concomitant GC dose, mg/day, PSL equivalent	0 (0; 5)		
Number of concomitant HCQ, *n* (%)	21 (10.0)		
Number of concomitant immunosuppressant use (%)	70 (33.2)		
MMF, *n* (%)	7 (3.3)		
AZA, *n* (%)	23 (10.9)		
TAC, *n* (%)	23 (10.9)		
CSA, *n* (%)	7 (3.3)		
MTX, *n* (%)	10 (4.7)		
MZR, *n* (%)	7 (3.3)		
BEL, *n* (%)	1 (1.0)		
SELENA-SLEDAI score	17.2±14.9		
BILAG organ-domain involvement of at least 1A or 2B, *n* (%)	140 (66.4)		
BILAG organ-domain involvement by category, *n* (%) A or B domain scores only			
General	67 (31.8)		
Mucocutaneous	90 (42.7)		
Neurological	46 (21.8)		
Musculoskeletal	30 (14.2)		
Cardiovascular and respiratory	28 (13.3)		
Renal	81 (38.4)		
Haematology	44 (20.9)		
WBC (/μL)	4900±2600		
Lymphocyte (/μL)	960±645		
CRP (mg/dL)	0.76±1.82		
ESR (mm/h)	46±31		
Serum IgG (mg/dL)	1700±663		
Anti-dsDNA antibody (U/mL)	89.9±224.5		
Anti-Sm antibody (U/mL)	46.4±128.5		
CH_50_ (U/mL)	37.3±17.4		

Data represent mean ± standard deviation, median (IQR) or number (%) of patients.

Statistical analysis was performed using the Mann–Whitney U test or Pearson's χ2 test.

APSAntiphospholipid antibody syndromeAZAazathioprineBELbelimumabBILAGBritish Isles Lupus Assessment Group IndexCH_50_50% haemolytic unit of complementCRPC reactive proteinCSAcyclosporin AdsDNAdouble-stranded DNAESRerythrocyte sedimentation rateGCglucocorticoidHCQhydroxychloroquineHCshealthy controlsMMFmycophenolate mofetilMTXmethotrexateMZRmizoribinePSLprednisoloneSELENA-SLEDAISafety of Estrogens in Lupus Erythematosus National Assessment SLE Disease Activity IndexSLEsystemic lupus erythematosusTACtacrolimusTMAthrombotic microangiopathyWBCwhite blood cell

In this study, laboratory data such as WBC and lymphocyte counts in healthy subjects were not gathered. They were reported as 4400/µL of WBCs and 2100/µL of lymphocytes in healthy subjects of similar age and sex,^26^ and lymphocytes in patients with SLE tended to be low.

### Significant increase in activated CD8^+^ T (especially type 1 CD8^+^ T (Tc1)) cells in peripheral blood samples of patients with SLE

Immunophenotyping of peripheral blood samples from 211 patients with SLE and 48 HCs was performed to investigate the immunological characteristics of patients with SLE. A comparison of the percentage of each immunophenotype among PBMCs revealed significantly increased naïve CD8^+^ T cells, terminally differentiated CD8^+^ T cells (T_EMRA_), activated CD8^+^ T cells (including both Tc1 and type 17 CD8^+^ T cell (Tc17) subsets), activated CD4^+^ T cells, double-negative B cells, plasmocytes and monocytes in patients with SLE than those in HCs (p<0.05; figure 1). In particular, activated CD8^+^ T and Tc1 cells were markedly increased, as were plasmocytes, which are an important contributor in the SLE pathology (activated CD8^+^ T cells: 4.43% in SLE and 1.06% in HC; activated Tc1: 1.35% in SLE and 0.49% in HC and plasmocytes: 1.39% in SLE and 0.32% in HC). Similarly, activated CD8^+^ T cells, activated Tc1 cells and plasmocytes were markedly increased in 90 treatment-naïve patients with SLE compared with those in HCs (online supplemental figure 3).

Subsequently, the function of activated CD8^+^ T cells, which were increased in peripheral blood samples, was evaluated by flow cytometry. There was significantly greater production of granzyme B, perforin and IFN-γ in activated CD8^+^ T cells of patients with SLE than that in HCs after comparing eight treatment-naïve patients with SLE and age- and sex-matched HCs (p<0.05; figure 1B,C). Thus, activated CD8^+^ T cells tended to have higher cytotoxicity and greater inflammatory ability in patients with SLE than those in HCs.

### Correlation of activated CD8^+^ T cells in the peripheral blood of patients with SLE to clinical findings and increases in systemic, renal and haematological involvement

**Figure 2:**
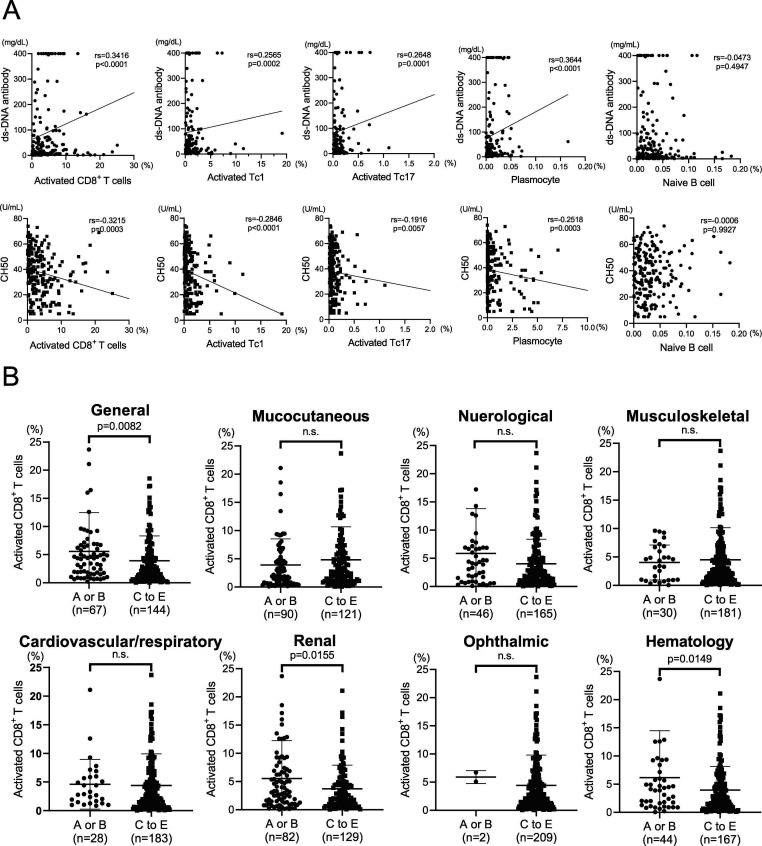
Association between clinical findings and CD8^+^ T cells in patients with SLE. (**A**) Relationship between activated CD8^+^ T cells, activated type 1 CD8^+^ T cells (Tc1), activated type 17 CD8^+^ T cells (Tc17), plasmocytes or naïve B cells, and dsDNA antibody or 50% haemolytic unit of complement (CH_50_) in patients with SLE. Spearman’s rank correlation coefficient. rs=coefficient. (**B**) Comparison of activated CD8^+^ T cells between active organ involvement and no active organ involvement in each subcohort. Values represent mean±SEM student’s t-test. dsDNA, double-stranded DNA; SLE, -stranded DNA; SLE, systemic lupus erythematosus.

The association between clinical findings of SLE and activated CD8^+^ T cells was examined. As with plasmocytes, the percentages of activated CD8^+^ T, activated Tc1 and activated Tc17 cells correlated positively with anti-dsDNA antibody titres and negatively with CH_50_. The correlation between activated Tc17 cells and CH_50_ was weaker (figure 2).

Subsequently, the association between organ involvement of SLE and activated CD8^+^ T cells was examined. The percentages of activated CD8^+^ T cells were compared between patients with active disease who met the BILAG index A or B organ-domain involvement criteria and those with inactive disease who met BILAG index C–E organ-domain involvement. Activated CD8^+^ T cells were significantly increased in patients with active systemic, renal and haematological organ involvement (figure 2B).

A similar analysis in 90 treatment-naïve patients with SLE showed that the proportions of activated CD8^+^ T cells, activated Tc1 cells and plasmocytes correlated significantly with the anti-dsDNA antibody titre and CH_50_, and that activated CD8^+^ T cell numbers significantly increased in patients with highly active systemic, neurological and renal involvement (online supplemental figure 4).

### Classification of patients with SLE according to the differentiated subsets of CD8^+^ T cells

**Figure 3:**
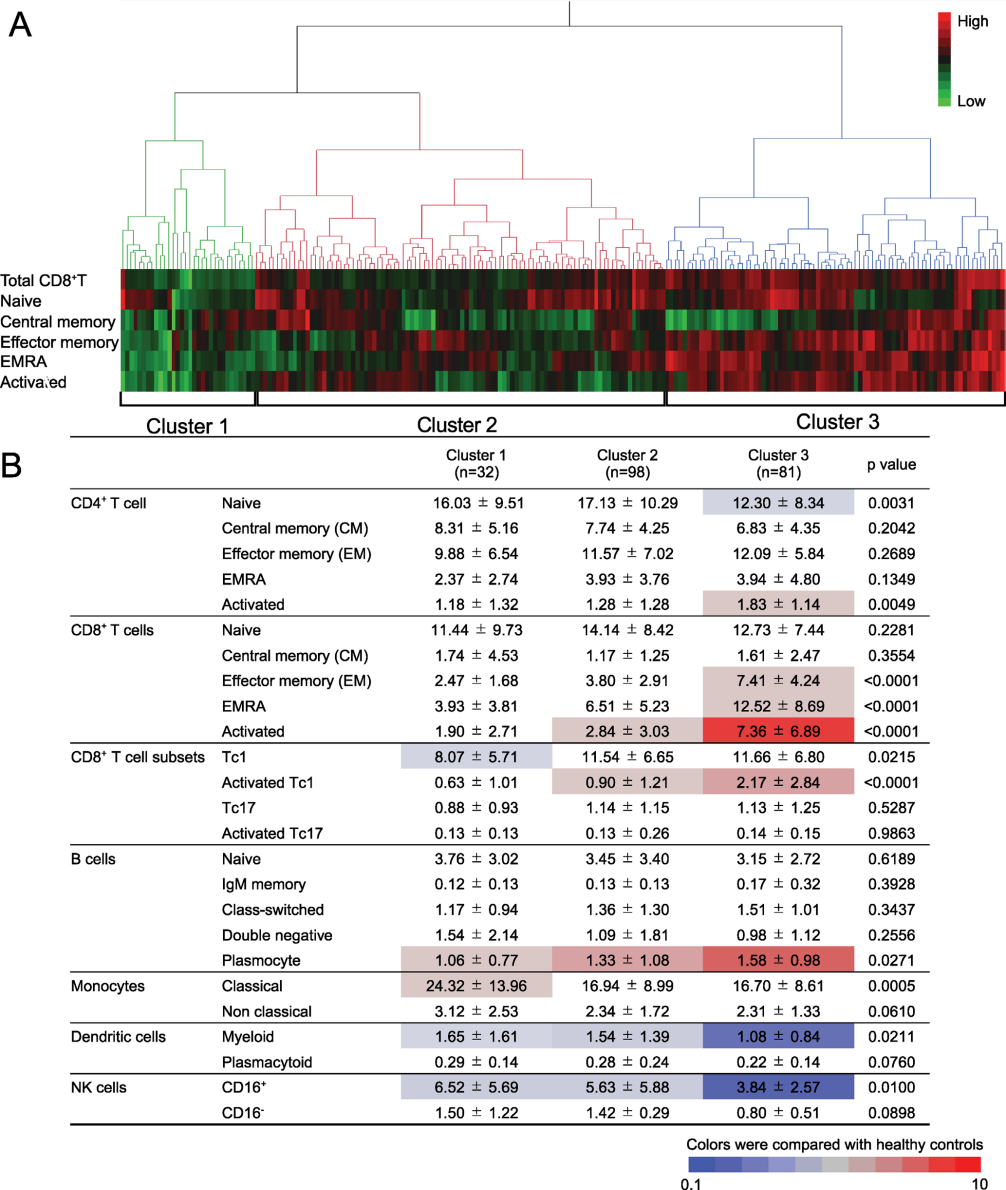
Stratification of patients with SLE by differentiated subsets of CD8^+^ T cells. (**A**) Hierarchical statistical clustering of patients with lupus in six items using Ward’s method. (**B**) Differences in the baseline percentage of peripheral blood lymphocyte subsets for individual patients in the three CD8 subgroups. Values representing mean±SEM p values in the univariate analysis were determined using one-way analysis of variance. IgM, immunoglobulin M; NK, natural killer; SLE, systemic lupus erythematosus.

As SLE is an immunologically heterogeneous disease, and a strong association between CD8 cells and clinical findings has been demonstrated in some patients, we performed cluster analysis of patients with SLE based on differentiation phenotyping of peripheral CD8^+^ T cells and divided the patients into three subgroups (figure 3A). Cluster 1 included patients with overall lower percentages of CD8^+^ T cell subsets, Cluster 2 included patients with a higher percentage of naïve CD8^+^ T cells and Cluster 3 included patients with higher percentages of the effector memory subset, the CD8^+^ T_EMRA_ subset and activated CD8^+^ T cells. Furthermore, when other immunophenotypes were compared among these three subgroups, activated CD4^+^ T cells and plasmocytes, which are a characteristic feature of patients with SLE compared with HCs, were significantly higher in Cluster 3, which comprised patients with severe immune abnormalities (figure 3B).

The baseline clinical findings of the three subgroups are shown in table 2. Although there was no difference in age, sex, disease duration or treatment background, Cluster 3 had a higher proportion of patients with active organ involvement (the percentage of patients with at least one organ-domain lesion with BILAG index A or two organ-domain lesions with BILAG index B; 68.7% vs 56.1% vs 77.8%), significantly higher titres of anti-dsDNA and anti-Sm antibodies and significantly lower CH_50_ counts compared with Clusters 1 and 2. The proportions of patients requiring remission induction therapy tended to be higher in Clusters 1 and 3 than that in Cluster 2. Among the 211 patients with SLE, 90 patients who underwent renal biopsy were included in a comparison of pathological findings among the three subgroups. Cluster 3 had a higher proportion of patients with proliferative lupus nephritis of Class III or IV (± V), but no patient had Class V membranous nephropathy compared with Clusters 1 and 2 (online supplemental figure 5).

**Table 2:** Clinical characteristics in the CD8^+^ T cell three subgroups of patients with SLE

Baseline clinical findings	Cluster 1 (n=32)	Cluster 2 (n=98)	Cluster 3 (n=81)	P value
Average age	48.1±17.4	41.8±14.5	40.4±17.7	0.0735
Female, *n* (%)	27 (84.4)	90 (91.8)	72 (88.9)	0.4885
Disease duration (month)	40.0(3.0; 227.5)	37.5(4.5; 145.0)	68.0(3.0; 179.0)	0.5790
Treatment naive (%)	14 (43.8)	41 (41.8)	35 (43.2)	0.9740
Concomitant GC dose, mg/day, PSL equivalent	0.0(0.0; 5.0)	2.5(0.0; 5.0)	0.0(0.0; 5.0)	0.7199
Number of concomitant HCQ, *n* (%)	3 (9.4)	10 (10.2)	8 (9.9)	0.9903
Number of concomitant immunosuppressant use (%)	10 (31.3)	32 (32.7)	28 (34.6)	0.9340
MMF, *n* (%)	0 (0.0)	4 (4.08)	3 (3.7)	0.3070
AZA, *n* (%)	1 (3.1)	11 (11.2)	11 (13.6)	0.1960
TAC, *n* (%)	6 (18.8)	7 (7.1)	10 (12.3)	0.1754
CSA, *n* (%)	1 (3.1)	4 (4.1)	2 (2.5)	0.8314
MTX, *n* (%)	3 (9.4)	6 (6.1)	1 (1.2)	0.0967
MZR, *n* (%)	2 (6.3)	3 (3.1)	2 (2.5)	0.6360
BEL, *n* (%)	0 (0.0)	1 (1.0)	0 (0.0)	0.4632
SELENA-SLEDAI score	8.5(6.0; 14.3)	8.0(4.0; 14.3)	11.0(6.0; 19.5)	0.1117
BILAG organ-domain involvement of at least 1A or 2B, *n* (%)	22 (68.7)	55 (56.1)	63 (77.8)	0.0090
BILAG organ-domain involvement by category, *n* (%) A or B domain scores only				
General	11 (38.3)	25 (25.5)	31 (38.3)	0.1763
Mucocutaneous	14 (43.8)	43 (43.9)	33 (40.8)	0.9061
Neurological	5 (15.63)	21 (21.4)	20 (24.7)	0.5579
Musculoskeletal	8 (25.0)	13 (13.3)	9 (11.1)	0.1881
Cardiovascular and respiratory	6 (18.8)	13 (13.3)	9 (11.1)	0.5773
Renal	10 (31.6)	31 (31.6)	40 (49.4)	0.0354
Haematology	8 (25.0)	15 (15.3)	21 (25.9)	0.1753
WBC (/μL)	5036±2543	5829±2907	4463±2473	0.0366
Lymphocyte (/μL)	926±749	987±609	939±631	0.8414
CRP (mg/dL)	2.21±3.71	0.57±1.20	0.42±0.74	<0.0001
ESR (mm/h)	57±38	40±28	48±30	0.0213
Serum IgG (mg/dL)	1849.8±767.7	1580.6±613.3	1778.8±661.0	0.0502
Anti-dsDNA antibody (U/mL)	58.3±156.8	59.1±101.7	142.0±324.0	0.0312
Anti-Sm antibody (U/mL)	8.8±21.9	35.6±109.3	71.3±158.6	0.0325
CH_50_ (U/mL)	44.3±17.6	39.7±15.9	31.5±17.4	0.0002
Induction therapy, *n* (%)	62.5	43.9	65.4	0.0100
High-dose GC+IVCY	25	25.5	24.7	
High-dose GC+MMF	0	6.1	18.5	
High-dose GC+RTX	9.4	3.1	10.4	
High-dose GC+others	28.1	9.2	12.3	

APS, antiphospholipid antibody syndrome; AZA, azathioprine; BEL, belimumab; BILAG, British Isles Lupus Assessment Group Index; CH_50_, 50% haemolytic unit of complement; CRP, C reactive protein; CSA, ciclosporin A; dsDNA, double-stranded DNA; ESR, erythrocyte sedimentation rate; GC, glucocorticoid; HCQ, hydroxychloroquine; IgGimmunoglobulin GMMF, mycophenolate mofetil; MTX, methotrexate; MZR, mizoribine; PSL, prednisolone; SELENA-SLEDAI, Safety of Estrogens in Lupus Erythematosus National Assessment SLE Disease Activity Index; SLE, systemic lupus erythematosus; TAC, tacrolimus; TMA, thrombotic microangiopathy; WBC, white blood cell

### Association of baseline CD8^+^ T cell activation with treatment resistance after 24-week treatment

Clinical signs were compared between the subgroups of 115 patients with SLE who were evaluated after 24 weeks. There was a lower proportion of patients who received low-dose glucocorticoids (PSL≤7.5 mg/day) after 24 weeks of treatment and the anti-dsDNA antibody titre was significantly higher in Cluster 3 (which included patients with severe baseline immune abnormalities) than that in Clusters 1 and 2 (table 3). Cluster 3 showed improvement in the activation of CD4^+^ T cells and abnormal differentiation of B cells, whereas CD8^+^ T cells remained activated after 24 weeks during the evaluation of each immune-cell subset (online supplemental figure 6). There was no significant difference between the 10 patients who achieved the definition of remission in SLE after 24 weeks and the 105 who did not, for each immunological subset after treatment (online supplemental figure 7). After 24 weeks, the SLICC/ACR damage index did not differ among the three groups and there was no association with immune subsets.

**Table 3:** Clinical characteristics at 24 weeks after treatment

	Cluster 1 (n=17)	Cluster 2 (n=51)	Cluster 3 (n=47)	P value
WBC (/μL)	7371±3924	6655±2458	7028±2935	0.6394
Lymphocyte (/μL)	831±531	1309±1336	1205±780	0.2649
CRP (mg/dL)	1.22±4.05	0.19±0.38	0.21±0.69	0.0604
ESR (mm/h)	23±16	20±18	27±26	0.3571
IgG (mg/dL)	939.7±354.4	1195.0±752.6	1146.7±638.6	0.6050
Anti-dsDNA antibody (U/mL)	3.7±4.3	15.9±35.6	22.9±62.7	0.0337
CH_50_ (U/mL)	51.8±14.5	51.4±12.4	49.1±15.5	0.7385
SELENA-SLEDAI score	0(0; 6)	0(0; 2)	2(0; 4)	0.4769
BILAG organ-domain involvement of at least 1A or 2B, *n* (%)	2 (11.8)	1 (2.0)	1 (2.1)	0.2357
BILAG organ-domain involvement by category, *n* (%) A or B domain scores only				
General	1 (5.9)	0 (0.0)	1 (2.1)	0.2361
Mucocutaneous	3 (17.7)	6 (11.8)	1 (2.1)	0.0645
Neurological	1 (5.9)	1 (2.0)	0 (0.0)	0.2564
Musculoskeletal	2 (11.8)	2 (3.9)	3 (6.4)	0.5360
Cardiovascular and respiratory	0 (0.0)	0 (0.0)	0 (0.0)	
Renal	1 (5.9)	4 (7.8)	6 (12.8)	0.6068
Haematology	1 (5.9)	1 (2.0)	2 (4.3)	0.6920
Concomitant GC dose, mg/day, PSL equivalent	7.5(0.0; 11.3)	6.0(0.0; 12.5)	10.0(5.0, 10.0)	0.4226
PSL≤7.5 mg/day (%)	10 (58.8)	28 (54.9)	15 (31.9)	0.0367
Number of concomitant HCQ, *n* (%)	7 (41.2)	21 (41.2)	20 (42.6)	0.9892
Number of concomitant immunosuppressant use, *n* (%)	7 (41.2)	31 (60.8)	33 (70.2)	0.1059
Average number of immunosuppressant	1(0; 1)	0(0;1)	1(0; 1)	0.1650
MMF, *n* (%)	3 (17.7)	10 (19.6)	11 (23.4)	0.8441
AZA, *n* (%)	2 (11.8)	7 (13.7)	10 (21.3)	0.5151
TAC, *n* (%)	2 (11.8)	5 (9.8)	4 (8.5)	0.9254
CSA, *n* (%)	0 (0.0)	1 (2.0)	0 (0.0)	0.4410
MTX, *n* (%)	1 (5.9)	5 (9.8)	2 (4.3)	0.5452
MZR, *n* (%)	0 (0.0)	0 (0.0)	0 (0.0)	
BEL, *n* (%)	1 (5.9)	5 (9.8)	6 (13.0)	0.6790
LLDAS attainment, *n* (%)	7 (41.2)	24 (47.1)	14 (29.8)	0.2085
DORIS remission attainment, *n* (%)	2 (11.8)	6 (11.7)	2 (4.26)	0.3415
SLICC/ACR damage index	0(0; 1)	0(0; 0)	0(0; 0)	0.9515

DORIS remission: definitions of remission in SLE.^40^

SLICC/ACR damage index: Systemic Lupus International Collaborating Clinics/American College of Rheumatology damage index.^41^

AZA, azathioprine; BEL, belimumab; BILAG, British Isles Lupus Assessment Group Index; CH_50_, 50% haemolytic unit of complement; CRP, C reactive protein; CSA, ciclosporin A; dsDNA, double-stranded DNA; ESR, erythrocyte sedimentation rate; GC, glucocorticoid; HCQ, hydroxychloroquine; IgGimmunoglobin GLLDAS, lupus low disease activity state; MMF, mycophenolate mofetil; MTX, methotrexate; MZR, mizoribine; PSL, prednisolone; SELENA-SLEDAI, Safety of Estrogens in Lupus Erythematosus National Assessment SLE Disease Activity Index; TAC, tacrolimus; WBC, white blood cell(table 3)

## Discussion

In recent years, single-cell RNA sequencing and genome analyses have focused on the importance of CD8^+^ T cells and the innate immune system in SLE pathology.^10 27 28^ This study investigated the association between the characteristics of peripheral CD8^+^ T cells and clinical findings in patients with SLE. Activated CD8^+^ T cells have a greater capacity to produce granzyme B, perforin and IFN-γ. These cells are significantly increased in the peripheral blood of patients with SLE, and they were associated with the presence or absence of clinical signs and organ damage. This result is similar to the characteristics of CD8^+^ T cells, which play a role in the tissue pathogenesis of SLE^10 11 27 28^ and is a valuable finding since pathological conditions of the tissues were evaluated using peripheral blood samples.

Previous reports on peripheral CD8^+^ T cells in SLE include several reports on enhanced CD8^+^ T cell function^29^,^31^ as well as impaired CD8^+^ T cell function due to increased CD38 expression or metabolic abnormalities.^32^,^35^ In this study, activated CD8^+^ T cells showed no evidence of exhaustion, such as reduced cytotoxic capacity or inflammatory cytokine production (cell surface components, such as programmed cell death protein-1 and cytotoxic T lymphocyte-associated protein-4, were not assessed). This feature is similar to CD8^+^ T cells in local organs in lupus nephritis.^27 28^ Wu *et al* reported an increase in granzyme K^+^ CD8^+^ T cells producing inflammatory cytokines, such as IFN-γ rather than cytotoxic CD8^+^ T cells in renal tissues in lupus nephritis. Granzyme K^+^ CD8^+^ T cells may induce the differentiation of atypical B cells via IFN-γ production.^28^ Although granzyme K was not examined in this study, activated CD8^+^ T cells are a subset involved in the pathogenesis of SLE due to their enhanced IFN-γ production. Unlike previous reports on peripheral CD8^+^ T cells, this study included the enrollment of a high percentage of patients with highly active SLE accompanied by active organ involvement at baseline. As immunophenotyping of peripheral blood may reflect pathological tissue conditions,^12^,^14^ the results of this study appear to reflect active pathological tissue conditions. This assumption is supported by similar results observed in 90 treatment-naïve patients.

Patients with SLE were classified into three subgroups that greatly varied in their differences in CD8^+^ T cell subsets and other immune-cell subsets and clinical findings according to the differentiation stages of CD8^+^ T cells. The disease activity of SLE was higher in cluster 3 with activated CD8^+^ T cells and the abnormalities of other immune-cell subsets were more severe. Moreover, activated CD8^+^ T cells were associated with glucocorticoid doses that were required after treatment as well as residual immunological abnormalities. The results of this study and previous studies suggest that activated CD8^+^ T cells with an effector function may be involved in the pathogenesis of SLE through the following two processes: (1) activated CD8^+^ T cells directly damage organs (such as renal tissues) through the production of granzyme B and other mediators^10^,^12^; and (2) they enhance the induction of differentiation (increased serum plasmocytes) and production of autoantibodies (increased anti-dsDNA antibody titre) in B cells, owing to IFN-γ production or release of autoantigens from damaged cells.^28^ In the future, abnormalities of peripheral CD8^+^ T cells may be used to predict treatment responses in and prognosis of SLE.

Notably, activated CD8^+^ T cells persisted in peripheral blood after 6 months of treatment. Although glucocorticoids are a mainstay drug for remission induction therapy in SLE,^36^ this study showed no decrease in CD8^+^CD38^+^ human leucocyte antigen-DR^+^ T cells in patients treated with high-dose glucocorticoid monotherapy (data not shown). In a previous study in mice, glucocorticoid administration did not decrease CD8^+^ T cells or impair their function.^37^ Thus, in cases of CD8^+^ T cell activation, mycophenolate mofetil and calcineurin inhibitors, which inhibit the synthesis of CD8^+^ T cells,^38 39^ are expected to be effective, and new treatments targeting CD8^+^ T cells are required.

The limitations of this study include the fact that we did not compare CD8^+^ T cells in the peripheral blood samples of the patients and those in damaged organs. Consequently, we could not clarify whether the cells in pre-existing damaged organs are identical to those in peripheral CD8^+^ T cells. In future studies of patients with SLE whose peripheral lymphocytes are analysed with simultaneously obtained tissue biopsy specimens, we intend to evaluate CD8^+^ T cells in the tissue samples and investigate the association of peripheral CD8^+^ T cells with those in the tissues. Additionally, we performed each post-treatment evaluation or evaluation after 24 weeks in only 115 of the 211 patients. Therefore, the results were affected by selection bias. It is necessary to investigate the association between peripheral CD8^+^ T cells and treatment responses according to different treatments by thoroughly performing periodic evaluations since this study suggests the involvement of CD8^+^ T cells in treatment resistance. In addition, we could not examine how activated CD8^+^ T cells interact with or act on other immune cells. This requires further experimentation, such as coculturing activated CD8^+^ T cells with B cells in vitro.

In conclusion, the peripheral blood of patients with SLE contains activated CD8^+^ T cells, which are associated with disease activity and renal, haematological and other organ involvement (especially active lupus nephritis) and may be a potential predictor of residual immune abnormalities and difficulty in reducing therapeutic drug doses after treatment.

## supplementary material

10.1136/rmdopen-2024-005122 online supplemental file 1

10.1136/rmdopen-2024-005122 online supplemental file 2

10.1136/rmdopen-2024-005122 online supplemental file 3

10.1136/rmdopen-2024-005122 online supplemental file 4

10.1136/rmdopen-2024-005122 online supplemental file 5

10.1136/rmdopen-2024-005122 online supplemental file 6

10.1136/rmdopen-2024-005122 online supplemental file 7

10.1136/rmdopen-2024-005122 online supplemental file 8

## Data Availability

Data are available on reasonable request.
