# Supplementary figures and images for: Association of peripheral CD8+ T cell activation with disease activity and treatment resistance in systemic lupus erythematosus
Source: RMD Open. 2025 Feb 26;11(1):e005122. doi: 10.1136/rmdopen-2024-005122 (PMC11865784; doi:10.1136/rmdopen-2024-005122)

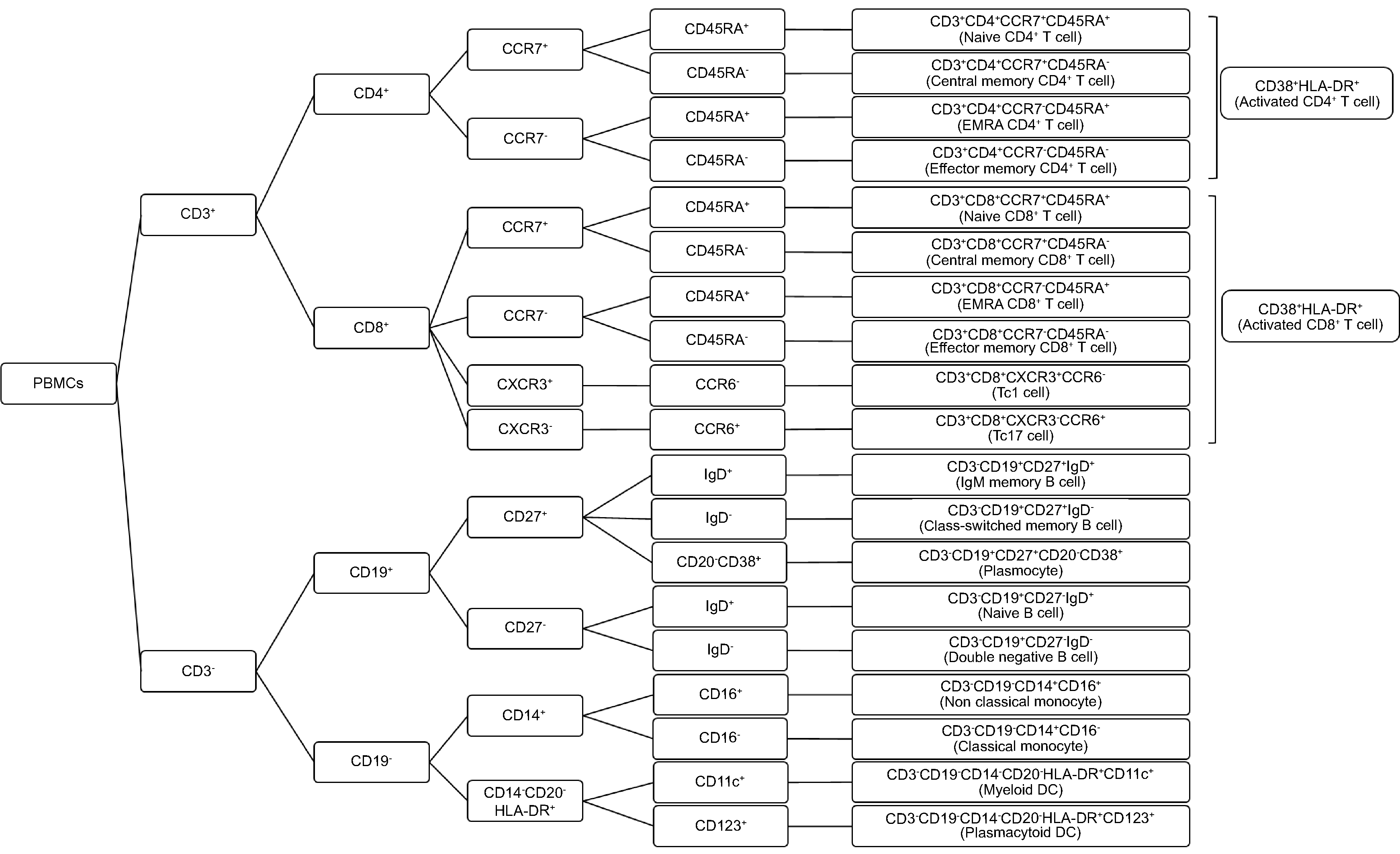

Supplement: online supplemental file 1 [file rmdopen-11-1-s001.tif]

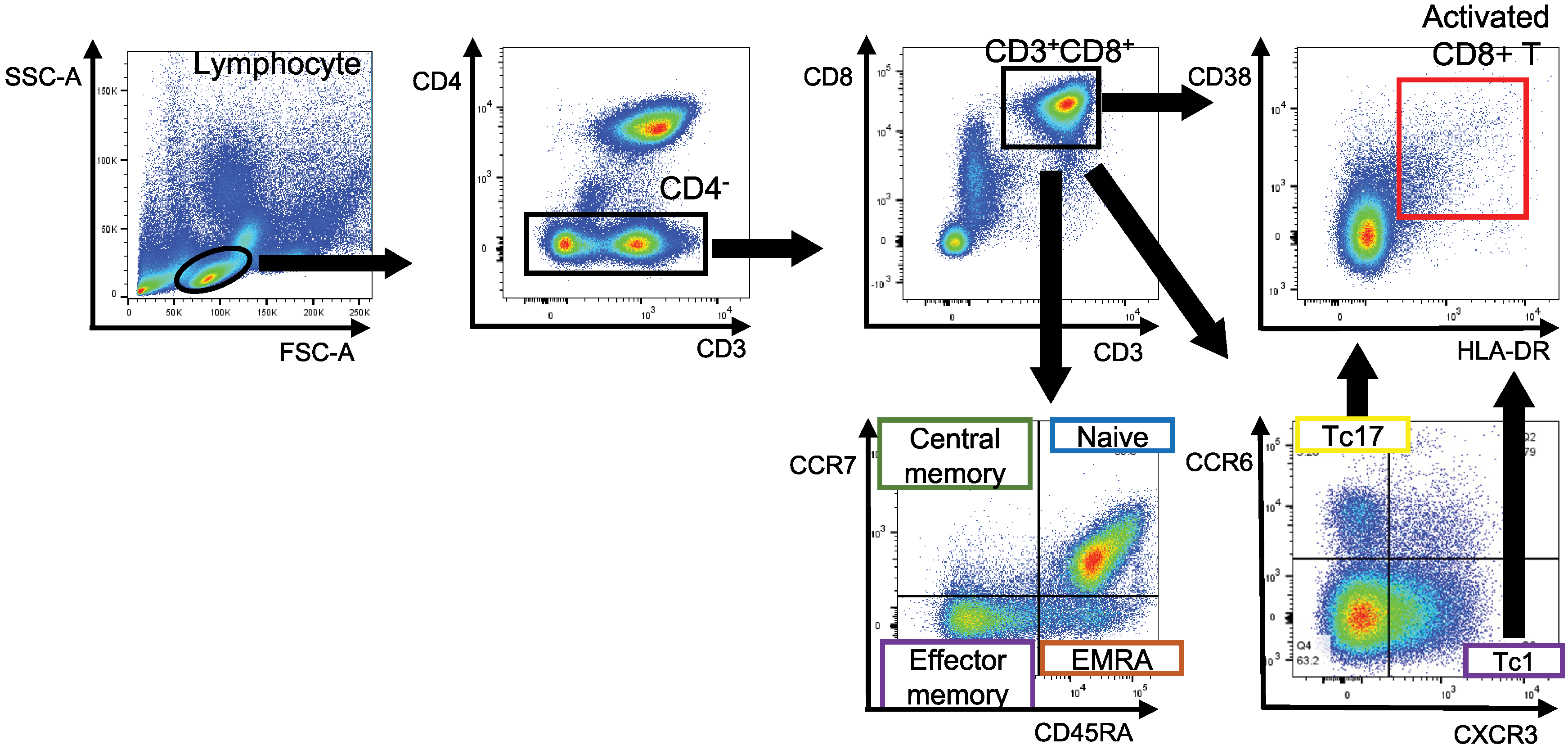

Supplement: online supplemental file 2 [file rmdopen-11-1-s002.tif]

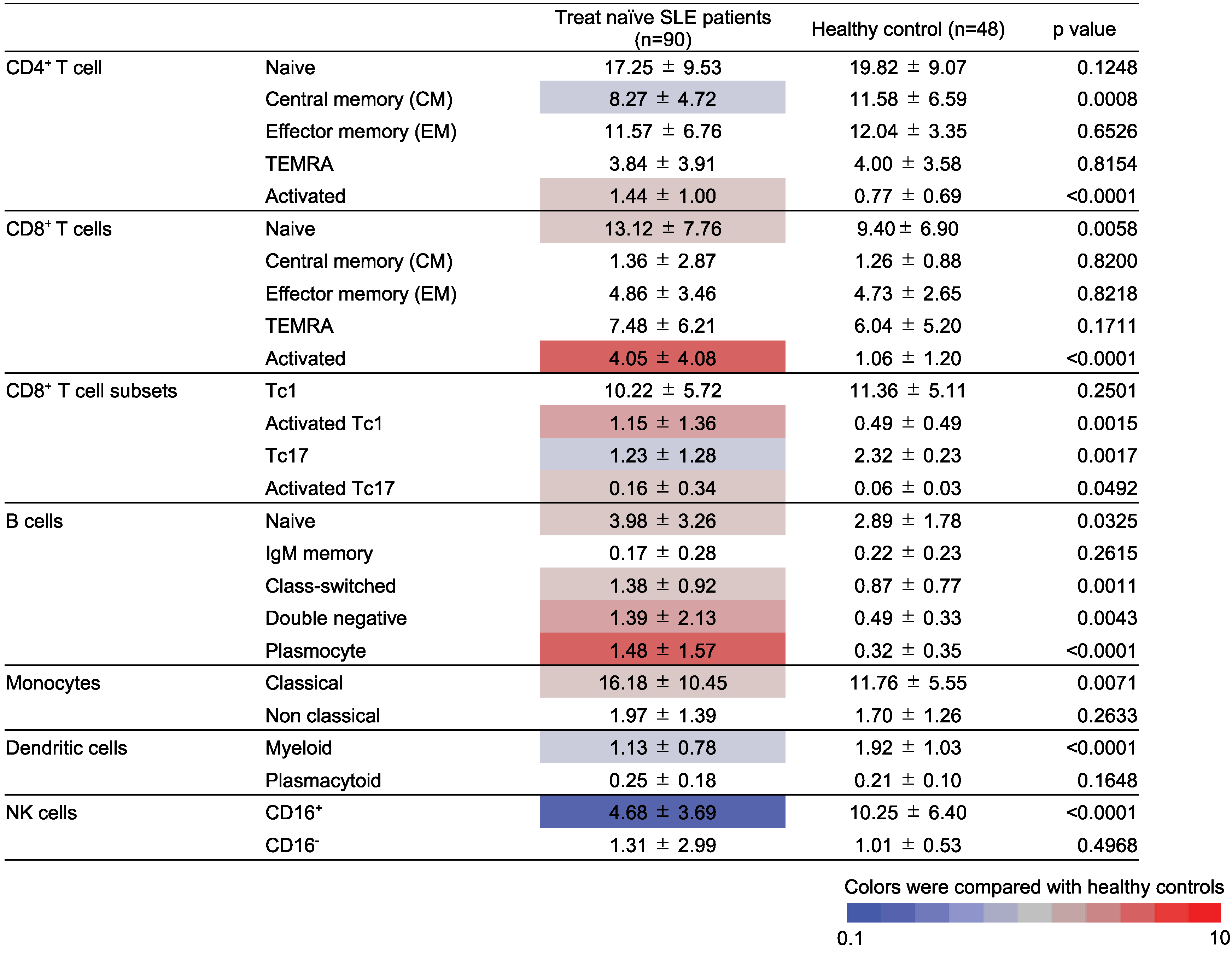

Supplement: online supplemental file 3 [file rmdopen-11-1-s003.tif]

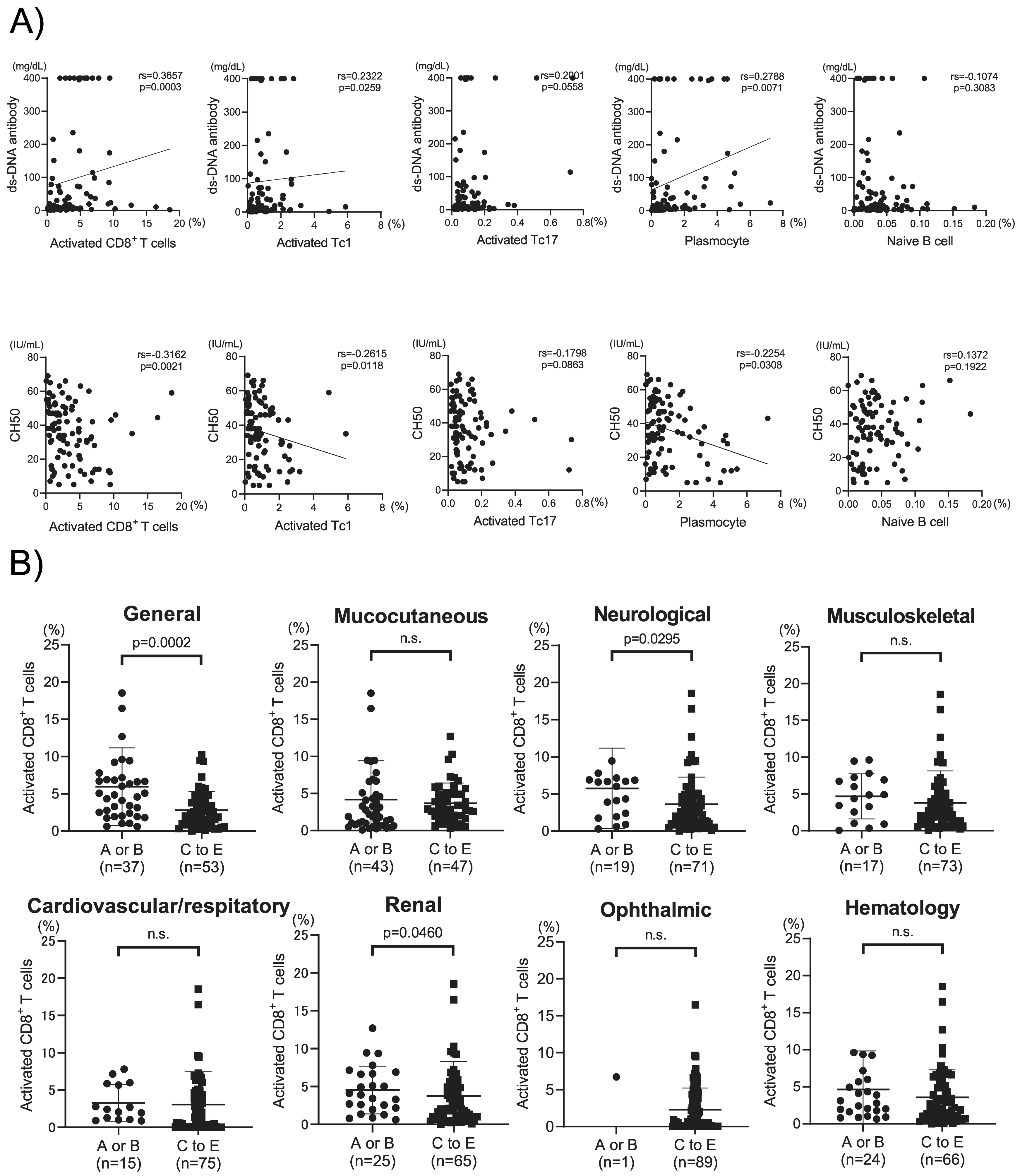

Supplement: online supplemental file 4 [file rmdopen-11-1-s004.tif]

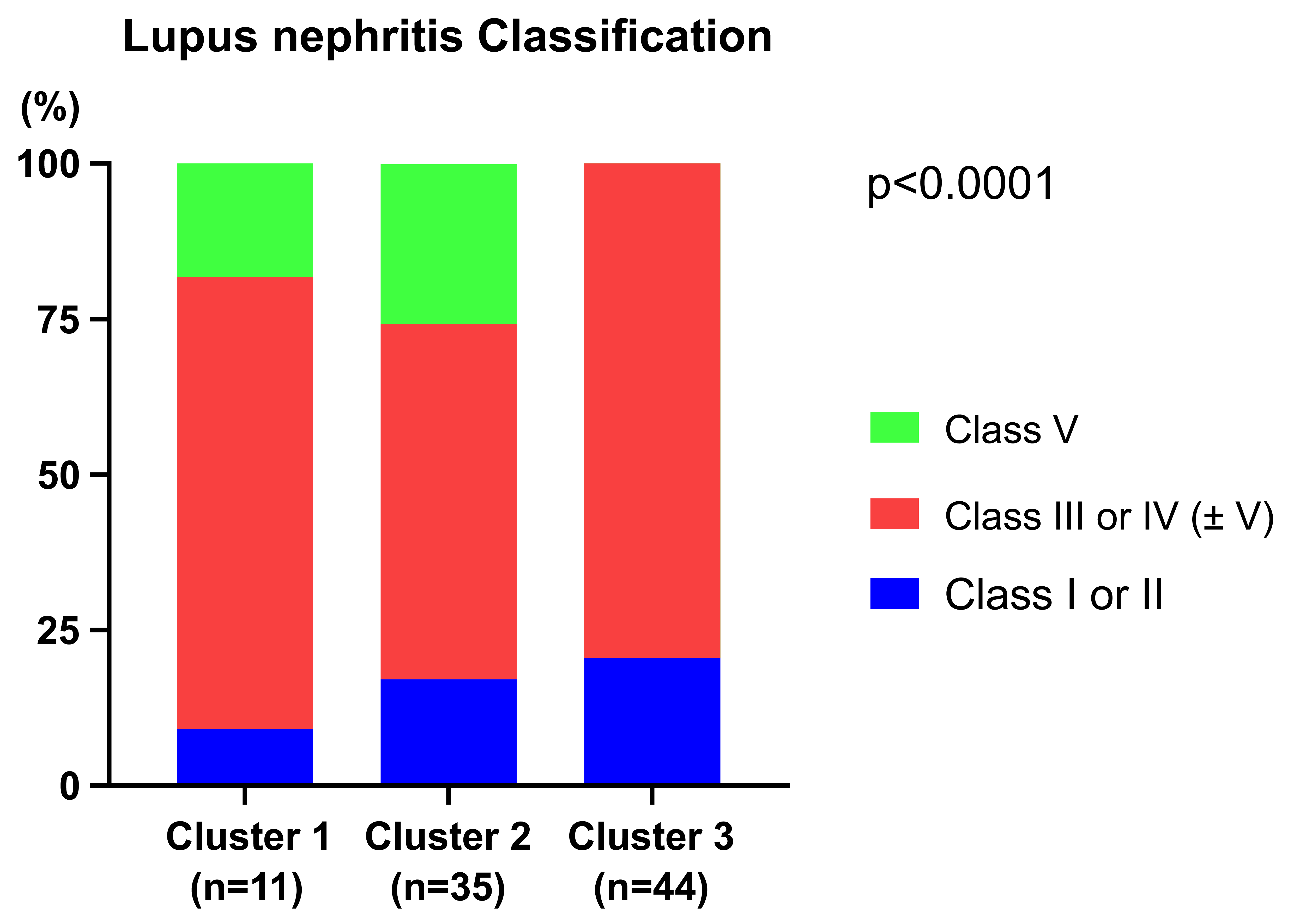

Supplement: online supplemental file 5 [file rmdopen-11-1-s005.tif]

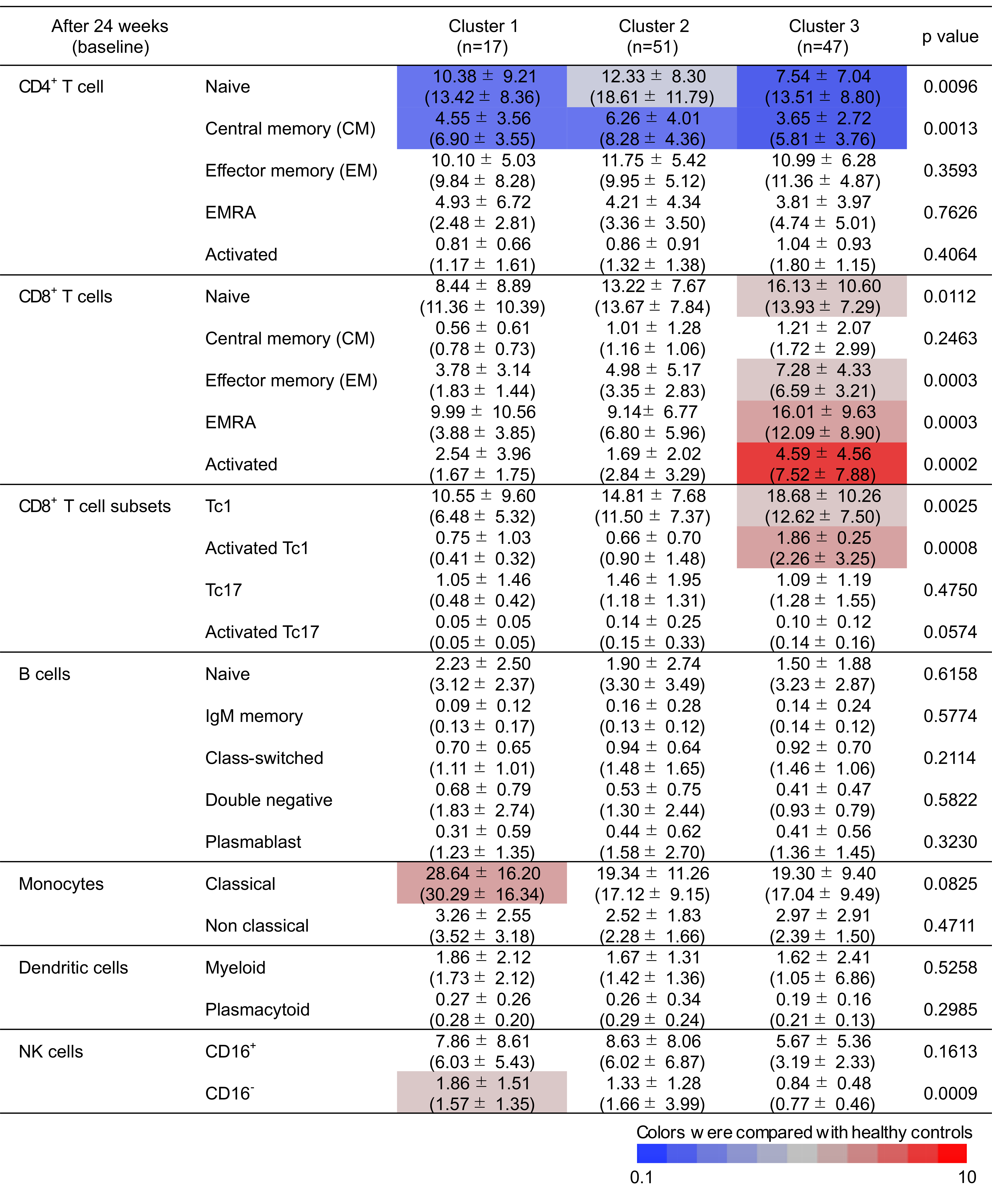

Supplement: online supplemental file 6 [file rmdopen-11-1-s006.tif]

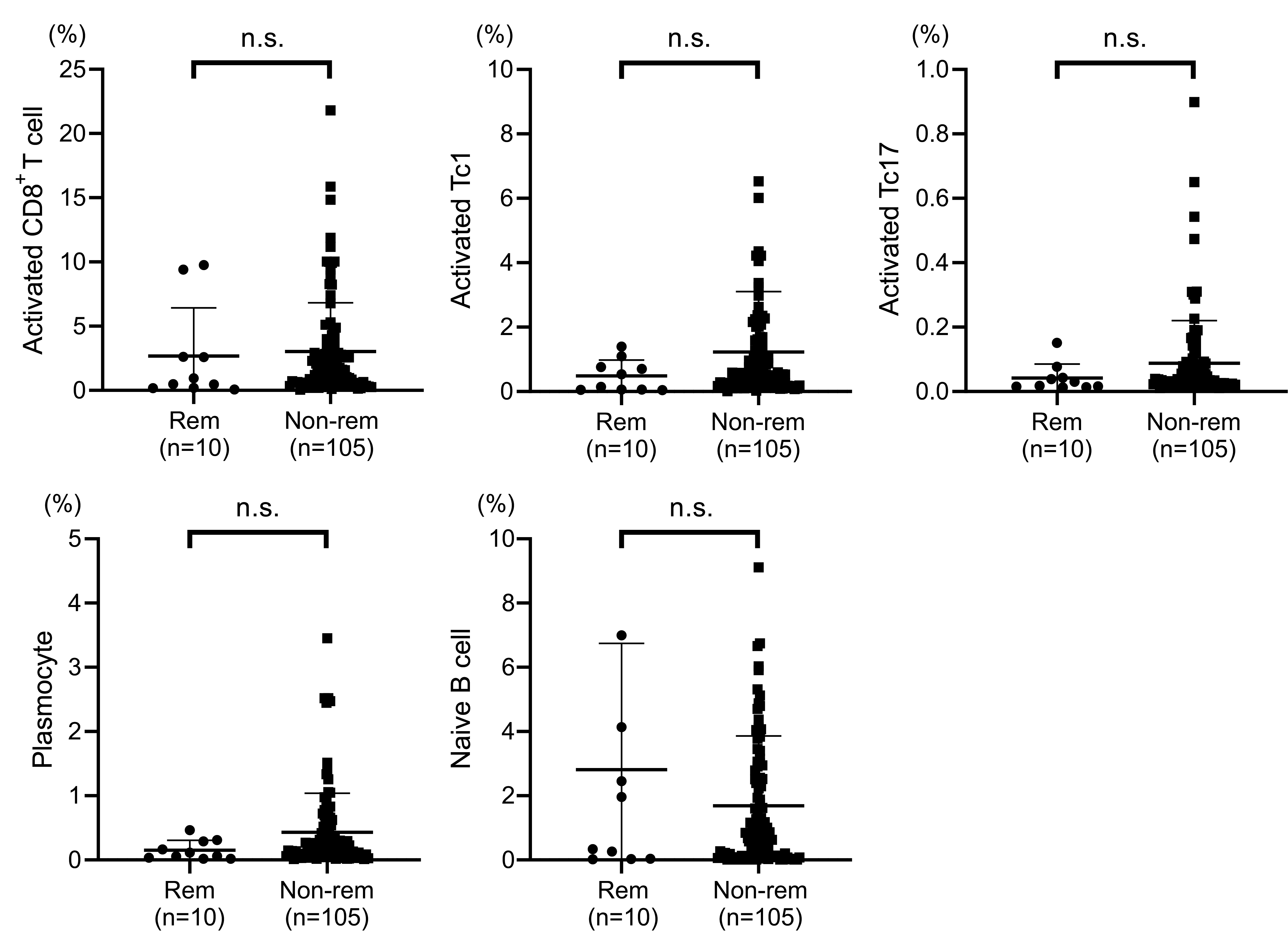

Supplement: online supplemental file 7 [file rmdopen-11-1-s007.tif]
