# Supplementary material for: Association of peripheral CD8+ T cell activation with disease activity and treatment resistance in systemic lupus erythematosus
Source: RMD Open. 2025 Feb 26;11(1):e005122. doi: 10.1136/rmdopen-2024-005122 (PMC11865784; doi:10.1136/rmdopen-2024-005122)
Supplement: online supplemental file 8 [file rmdopen-11-1-s008.docx]

**Supplemental Table 1**. List of antibodies used for flow cytometry

|  | | | |  |  |  |  |
| --- | --- | --- | --- | --- | --- | --- | --- |
| **Panels** | **T cells** | **T_reg_** | **Th1, Th2 Th17** | **B cells** | **DCs, monocytes, NK cells** | **T_reg_ 2** | **Tfh, Th1, Th2, Th17** |
| FITC | Live or dead | Live or dead | Live or dead | Live or dead | Live or dead | Live or dead | Live or dead |
|  | Invitrogen | Invitrogen | Invitrogen | Invitrogen | Invitrogen | Invitrogen | Invitrogen |
|  | L-23102 | L-23012 | L-23102 | L-23102 | L-23102 | L-23102 | L-23102 |
| PE | CCR7 | CD25 | CXCR3 | CD24 | CD56 | FoxP3 | CXCR5 |
|  | BD Pharmingen | BD Pharmingen | BD Pharmingen | BD Pharmingen |  | BD Pharmingen | R&D |
|  | 551773 | 555432 | 550633 | 555428 |  | 560082 | FAB190P |
| PerCP-Cy5.5 | CD4 | CD4 | CD4 | CD19 | CD123 | CD25 | CXCR3 |
|  | BD Pharmingen | BD Pharmingen | BD Pharmingen | BD Bioscience |  | BD Pharmingen | BD Pharmingen |
|  | 560650 | 560650 | 560650 | 340951 |  | 560503 | 560832 |
| PE-Cy7 | CD45RA | CCR4 | CCR6 | CD27 | CD11c | CD45RA | CCR6 |
|  | BD Pharmingen | BD Pharmingen | BD Pharmingen | eBioscience |  | BD Pharmingen | BD Pharmingen |
|  | 560675 | 561034 | 560620 | 25-0279-42 |  | 560675 | 560620 |
| APC | CD38 | CD127 | CD38 | CD38 | CD16 | CD127 | ICOS |
|  | BD Pharmingen | eBioscience | BD Pharmingen | BD Pharmingen |  | eBioscience | eBioscience |
|  | 560980 | 17-1278-41 | 560980 | 560980 |  | 17-1278-41 | 17-9948-41 |
| APC-H7 | CD8 | CD45RO | CD8 | CD20 | CD3 | CD69 | CD69 |
|  | BD Pharmingen | BD Pharmingen | BD Pharmingen | BD Pharmingen |  | BD Pharmingen | BD Pharmingen |
|  | 561423 | 561137 | 561423 | 560853 |  | 560737 | 560737 |
| V450 | CD3 | CD3 | CD3 | CD3 | CD14 | CD3 | CD3 |
|  | BD Horizon | BD Horizon | BD Horizon | BD Horizon |  | BD Horizon | BD Horizon |
|  | 560366 | 560366 | 560366 | 560366 |  | 560366 | 560366 |
| V500 | HLA-DR | HLA-DR | HLA-DR | IgD | HLA-DR | CD4 | CD4 |
|  | BD Horizon | BD Horizon | BD Horizon | BD Horizon | BD Horizon | BD Horizon | BD Horizon |
|  | 561225 | 561225 | 561225 | 561490 | 561225 | 560769 | 560769 |
|  | | | |  |  |  |  |

Upper row; marker, middle row; company, lower row; tube ID.

APC: allophycocyanin; DC: dendritic cell; FITC:
fluorescein-5-isothiocyanate; NK: natural killer; PE, phycoerythrin; PerCP-Cy5.5: peridinin chlorophyll protein-cyanine5.5; Th, T helper cells; Tfh, T follicular helper cells; T_reg_, regulatory T cells.
